# Deciphering male influence in gynogenetic Pengze crucian carp (*Carassius auratus* var. *pengsenensis*): insights from Nanopore sequencing of structural variations

**DOI:** 10.3389/fgene.2024.1392110

**Published:** 2024-05-09

**Authors:** Qianhui Chen, Biyu Wu, Chao Li, Liyun Ding, Shiting Huang, Junjie Wang, Jun Zhao

**Affiliations:** ^1^ Guangzhou Key Laboratory of Subtropical Biodiversity and Biomonitoring, School of Life Sciences, South China Normal University, Guangzhou, China; ^2^ Jiangxi Fisheries Research Institute, Nanchang, China

**Keywords:** Pengze crucian carp, gynogenesis, Nanopore sequencing, structural variations, male genetic materia, genetic diversity

## Abstract

In this study, we investigate gynogenetic reproduction in Pengze Crucian Carp (*Carassius auratus* var. *pengsenensis*) using third-generation Nanopore sequencing to uncover structural variations (SVs) in offspring. Our objective was to understand the role of male genetic material in gynogenesis by examining the genomes of both parents and their offspring. We discovered a notable number of male-specific structural variations (MSSVs): 1,195 to 1,709 MSSVs in homologous offspring, accounting for approximately 0.52%–0.60% of their detected SVs, and 236 to 350 MSSVs in heterologous offspring, making up about 0.10%–0.13%. These results highlight the significant influence of male genetic material on the genetic composition of offspring, particularly in homologous pairs, challenging the traditional view of asexual reproduction. The gene annotation of MSSVs revealed their presence in critical gene regions, indicating potential functional impacts. Specifically, we found 5 MSSVs in the exonic regions of protein-coding genes in homologous offspring, suggesting possible direct effects on protein structure and function. Validation of an MSSV in the exonic region of the polyunsaturated fatty acid 5-lipoxygenase gene confirmed male genetic material transmission in some offspring. This study underscores the importance of further research on the genetic diversity and gynogenesis mechanisms, providing valuable insights for reproductive biology, aquaculture, and fostering innovation in biological research and aquaculture practices.

## 1 Introduction

Reproduction serves as a crucial natural mechanism allowing all living organisms to pass on their genetic material to their offspring. While most animals engage in sexual reproduction, a minority reproduce through asexual means (de Meeûs et al., 2007). Gynogenesis, a unique form of asexual reproduction, necessitates sperm stimulation for egg development into an embryo without incorporating the male’s genetic materia (Hubbs and Hubbs, 1932). This mode of reproduction is observed in specific fish species (Cherfas, 1966; Yang et al., 1992; Lampert and Schartl, 2008), among other organisms (Spolsky et al., 1992; D'Souza et al., 2006; Taylor et al., 2018; Sperling et al., 2023).

The Pengze Crucian Carp (*Carassius auratus* var. *pengsenensis*), a natural triploid species found in Pengze County, Jiangxi Province, China, plays a significant role in the region’s economy due to its gynogenetic reproduction (Yang et al., 1992). This reproduction mechanism allows the development of embryos from unfertilized eggs upon sperm stimulation, retaining desirable female traits, thus offering substantial economic and production advantages (Zhou et al., 2020; Xia, 2023). Despite predictions by Müller’s ratchet theory of potential species extinction due to the accumulation of deleterious mutations in asexually reproducing organisms (Kondrashov, 1982), this species thrives across China, demonstrating high fecundity and adaptability (Liu et al., 2017). Research indicates that incidental homologous recombination and gene conversion during oocyte maturation can prevent harmful mutation accumulation, thereby reducing the risk of extinction (Wang et al., 2022). Notably, gynogenetic offspring are not exact clones of the mother, as evidenced by diverse offspring traits and the presence of male offspring in various sperm-stimulated combinations (Shu et al., 2001; Zhao and Chen, 2003; Zhou et al., 2020; Ding et al., 2021; Shi et al., 2022). Studies have confirmed the inheritance of male genetic material in offspring (Zhang et al., 1999; Zhao et al., 2004a; Mao et al., 2020; Ding et al., 2021; Zhao et al., 2021), challenging previous assumptions about genetic uniformity. Although homologous and heterologous male pronuclei do not fuse with the female pronucleus, the interaction between them may facilitate male genetic material transfer (Yang, 2004; Zhao et al., 2021). On the contrary, in some studies of other gynogenetic fish, it has been found that offspring and their females exhibit a high degree of genetic homogeneity, meaning that there is similarity or uniformity in genetic characteristics between offspring and between offspring and their females. The consequences of this may include reduced genetic diversity and a decreased ability to adapt to environmental changes (Hou et al., 2016; Oral et al., 2017). The phenomenon requiring further investigation to elucidate the underlying mechanisms and impacts on offspring phenotype.

Nanopore resequencing facilitates the generation of long reads, typically ranging from 10 Kb to 20 Kb. When these reads are aligned with a reference genome, they enable precise identification of genetic variations between the sample and the reference genome or among the samples themselves. Due to the extended length of these reads, this method is particularly useful for detecting structural variations (SVs) (Cretu Stancu et al., 2017), including insertions (INS), deletions (DEL), inversions (INV), tandem duplications (DUP), intrachromosomal or interchromosomal translocations (TRA), copy number variations (CNV), and other complex alterations of sequences longer than 50 bp (Alkan et al., 2011). These variations, which are numerous and contribute significantly to the complexity of the genome, provide a genetic basis for species adaptation to environmental changes (Carvalho and Lupski, 2016). They arise through various mechanisms, such as unequal recombination, replication slippage, and mobile element-mediated recombination. Compared to single nucleotide polymorphisms (SNPs), SVs encompass larger genomic regions and offer more complex genetic information. As vital genetic markers, SVs play a crucial role in regulating gene expression, enhancing crop traits, and investigating crop adaptability (Pokrovac and Pezer, 2022; Liu et al., 2023).

**FIGURE 1:**
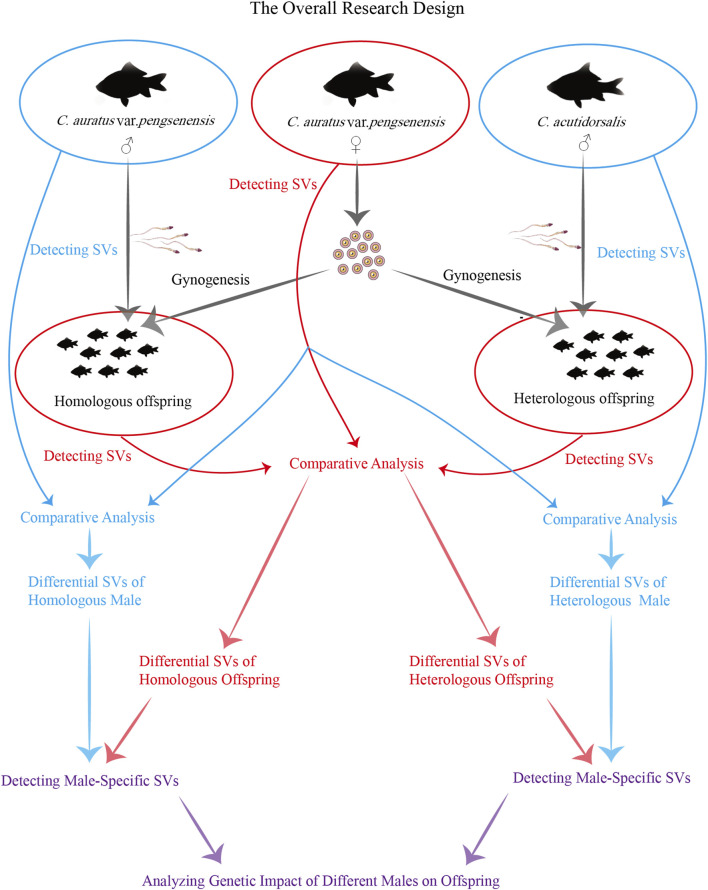
The ovreall research design.

Utilizing third-generation long-read sequencing technologies for resequencing the whole genomes of parents, along with both homologous (the male is from the same species) and heterologous (the male is from a different species) offspring, enables the detailed detection of SVs, which serve as crucial genetic markers. This advanced approach is pivotal for delineating the genetic discrepancies between homologous and heterologous offspring of Pengze Crucian Carp, as well as elucidating the male genetic contributions, providing essential insights into the complex mechanisms underpinning gynogenesis in triploid crucian carp (Figure 1). Furthermore, this study not only enriches our genomic knowledge base but also offers practical guidance for breeders aiming at breeding improvements and the development of new gynogenetic triploid crucian carp varieties. By exploring the intricate genetic landscape and reproductive modalities of this species, our research aims to contribute significantly to the field of reproductive biology, enhancing our understanding of reproductive diversity and informing future genetic and breeding strategies.

## 2 Materials and methods

### 2.1 Artificial breeding

For this experiment, we selected a female Pengze crucian carp, a male Pengze crucian carp, and a male sea carp (*Cyprinus acutidorsalis*), all of which were artificially selected from Guangzhou Jianbo Fish Farm Co., Ltd., as our breeding stock. Artificial insemination was performed on 13 March 2023. Artificial insemination was conducted on 13 March 2023. The female’s eggs were evenly divided into two portions, each fertilized separately with the sperm from the male Pengze crucian carp and the male sea carp. The fertilized eggs from the homologous and heterologous groups were evenly distributed onto hatching net plates and placed in two concrete hatching pools, each measuring 6 m × 4 m, within the same room. These pools were equipped with a circulating water system, and the water temperature was maintained between 24.5°C and 25.8°C with continuous aeration for 24 h. After hatching, the fry were initially fed boiled egg yolk for 3 days. Once the fry reached a length of 3-4 cm, we randomly selected 100 individuals from each group and transferred them to two separate circular tanks, each with a diameter of 3 m and a height of 1.5 m, for breeding. The daily diet for these fish consisted of feed containing 38% protein.

### 2.2 Sample collection

Parental muscle tissue samples from two Pengze crucian carps and one male sea carp were collected on 13 March 2023. The samples were immediately placed into microcentrifuge tubes pre-cooled with liquid nitrogen and then stored at −80°C for preservation. The parental samples were labeled as follows: PF (female Pengze crucian carp), PM (male Pengze crucian carp), and CM (male sea carp). On 18 September 2023, nine homologous offspring and nine heterologous offspring were randomly selected for the same sampling procedure. The samples from the homologous offspring were designated as PP1, PP2, PP3, PP4, PP5, PP6, PP7, PP8, and PP9, while those from the heterologous offspring were labeled as PC1, PC2, PC3, PC4, PC5, PC6, PC7, PC8, and PC9.

### 2.3 Third-generation Nanopore sequencing

On 19 September 2023, 21 frozen samples were sent to Wuhan Grandomics Biosciences Co., Ltd. (https://www.grandomics.com/) for third-generation Nanopore resequencing. Samples were collected, and high molecular weight genomic DNA was prepared by the CTAB method and followed by purification with QIAGEN^®^ Genomic kit (Cat#13343, QIAGEN) for regular sequencing, according to the standard operating procedure provided by the manufacturer. After the sample was qualified, size-select of long DNA fragments were performed using the PippinHT system (Sage Science, United States). Next, the ends of DNA fragments were repaired, and A-ligation reaction were conducted with NEBNext Ultra II End Repair/dA-tailing Kit (Cat# E7546). The adapter in the SQK-LSK109 (Oxford Nanopore Technologies, UK) was used for further ligation reaction and DNA liabrary was measured by Qubit^®^ 4.0 Fluorometer (Invitrogen, United States). About 700 ng DNA library was constructed and performed on a Nanopore PromethION sequencer instrument (Oxford Nanopore Technologies, UK) at the Genome Center of Grandomics (Wuhan, China).

### 2.4 WGS data clean and alignment

Raw data from Nanopore sequencing were base-called and converted into the fastq format using Drado v0.3.4 (https://github.com/nanoporetech/dorado) for subsequent analysis. The fastq data of the 21 samples underwent adapter and low-quality sequence removal using Porechop v0.2.4 (Bonenfant et al., 2023). Subsequently, with Nanofilt v2.8.0 (De Coster et al., 2018), we trimmed 90 bp from the beginning and 80 bp from the end of each read, filtering out reads shorter than 500 bp or with a quality score below 7. The cleaned data from each sample were aligned to the published triploid *Carassius gibelio* genome (NCBI RefSeq assembly: GCA_023724105.1) using Minimap2 v2.2.6 (Li, 2018) with default parameters. Alignment information for each sample was assessed using samtools v1.19 (Danecek et al., 2021) and mosdepth v0.3.4 (Pedersen and Quinlan, 2018).

### 2.5 Whole-genome detection of structural variations (SVs)

To ensure ensure subsequent differential analysis between females and males as well as the offspring genomes, a specific strategy for SV detection was employed (Supplementary Figure S1):

1. SVs in the PF data were detected using default parameters of Sniffles2 v2.2 (Smolka et al., 2024), cuteSV v2.0.3 (Jiang et al., 2022), and SVIM v2.0.0 (Heller and Vingron, 2019). The results from these three tools were compiled to create a composite dataset of PF’s SVs.
2. For males and offspring genomes, SV detection was exclusively carried out using Sniffles2 v2.2. The raw SV result files for each sample were further filtered using bcftools v.1.19 (Danecek et al., 2021) and a Python script to exclude SVs with “QUAL<20,” “SUPPORT<4,” “IMPRECISE” tags, and genotypes “*./.*” and “*0/0*”

Only DEL, INS, INV, and DUP were analyzed.

### 2.6 Detection of differential SVs (DSVs) and male-specific SVs (MSSVs) in offspring

To detect DSVs between PF and offsprings, as well as PF and males, the following approach was adopted. Firstly, SVs from males and offspring, filtered earlier, were merged with SV results of PF using SURVIVOR v1.0.6 (Jeffares et al., 2017). The parameters set for this were: “SURVIVOR merge sample.files 1,000 2 1 1 0 50 output.vcf.” If males and offspring SVs could be merged with PF’s SVs, these were eliminated using a Python script. This process was repeated until no consistent SVs with PF were detectable. The remaining DSVs were then screened to exclude those with read coverage in PF less than 4 in the DSV region. This resulted in the identification of genuine and significant DSVs between PF and offspring, and between PF and males.

For accurate and effective detection of MSSVs in offspring, the approach first involved excluding DSVs larger than 10 Kb. Then, SURVIVOR was used to detect MSSVs by merging homologous offsprings with the the homologous male and heterologous offsprings with the heterologous male, using the parameters: “SURVIVOR merge sample.files 100 2 1 1 0 50 output.vcf.” Subsequently, a separate analysis was conducted on both sets of nine homologous and heterologous offspring to identify common MSSVs in each group, with the parameters: “SURVIVOR merge sample.files 100 9 1 1 0 50 output.vcf.”

### 2.7 Gene annotation of MSSVs in offspring

Gene annotation for MSSVs detected in each offspring sample was carried out using ANNOVAR v2020.06.07 (Wang et al., 2010). This process was aimed at understanding the potential impacts of these variations. A local database was established using the annotation database corresponding to the crucian carp reference genome, and the MSSVs in offspring were annotated using the default parameters.

### 2.8 Validation of a specific MSSV

To validate MSSVs in offsprings, a focus was placed on a specific MSSV within the exonic regions of protein-coding genes that could cause frameshift mutations. Using samtools v1.19, reads from the bam alignment file that encompass the specified region were extracted and saved as a new bam file. This was followed by visualization using Geneious Prime v2022.2.2 (Deng et al., 2020), for further analysis. Primers for PCR validation of the MSSV were designed using Primer3 v2.3.7 (Untergasser et al., 2012). The DNA template with primers underwent a series of thermal cycles: initial denaturation at 95°C for 5 min, followed by 30 cycles of amplification(denaturation at 94°C for 30 s, annealing at 56°C for 45 s, and extension at 72°C for 30 s), and a final extension phase at 72°C for 10 min.

## 3 Results

### 3.1 Calling and analysis of SVs

**FIGURE 2:**
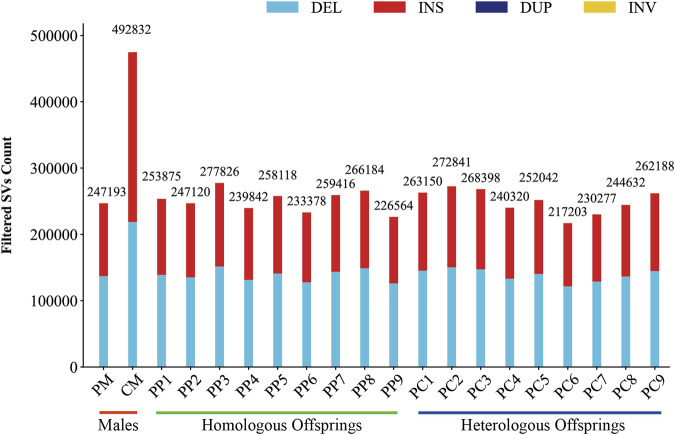
Filtered SVs counts in males, homologous, and heterologous offspring.

**FIGURE 3:**
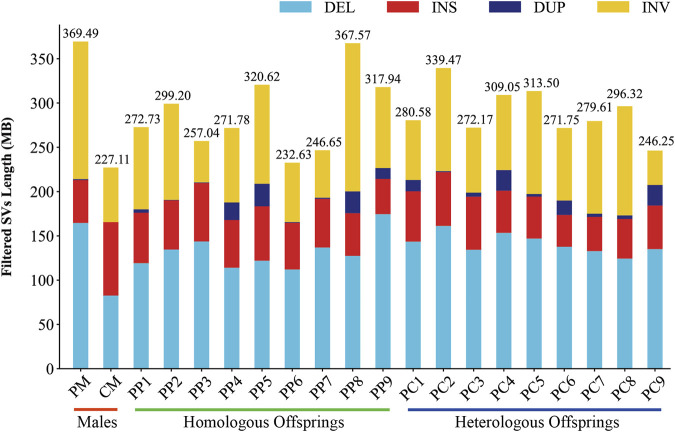
Filtered SVs lengths in males, homologous, and heterologous offspring.

**FIGURE 4:**
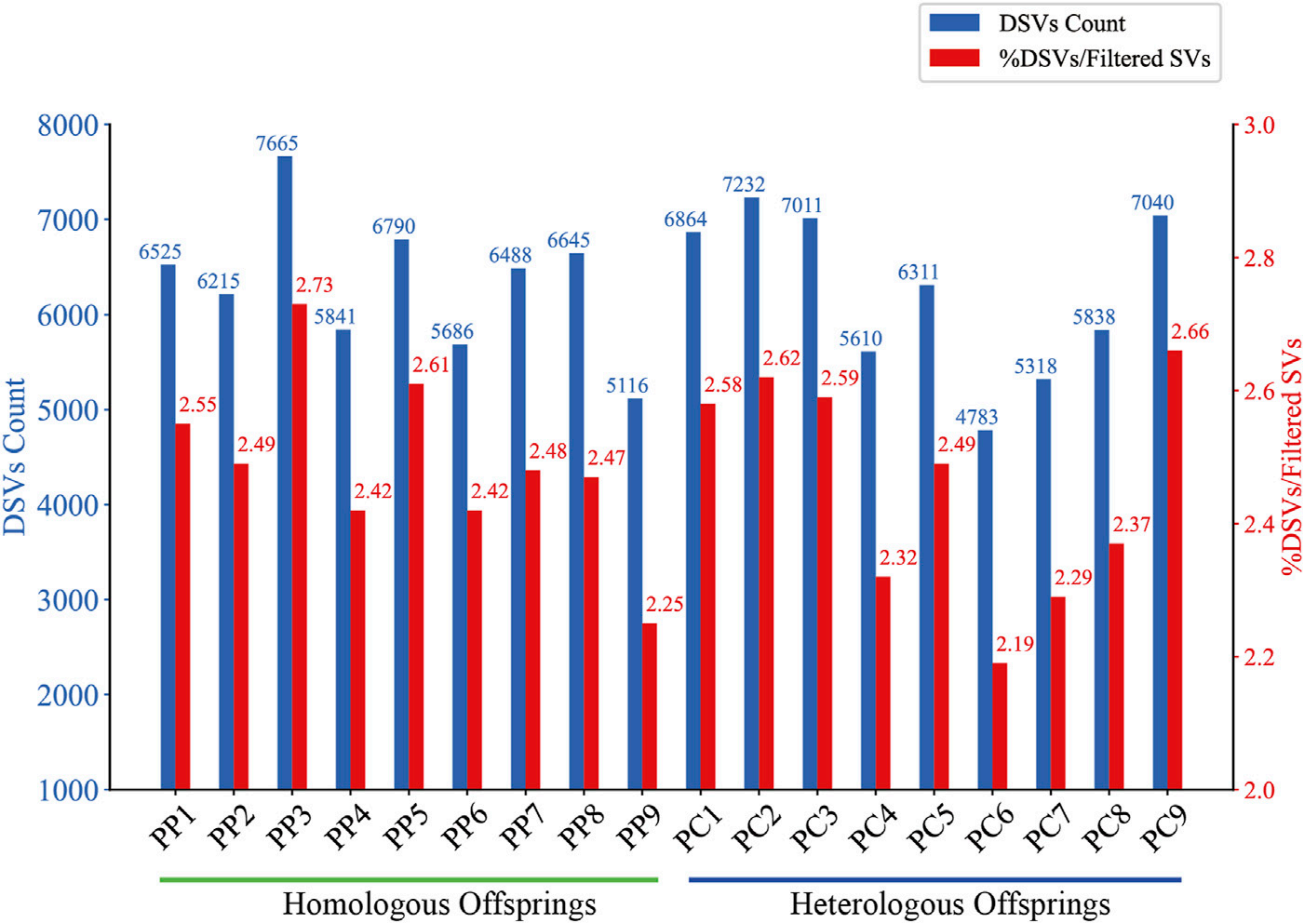
DSVs Count and Proportion in Homologous and Heterologous Offspring. The bar chart compares the absolute count of DSVs (blue bars) identified in homologous and heterologous offspring. The red bars represent the percentage of these DSVs relative to the total filtered SVs for each offspring.

In this investigation, we employed third-generation Nanopore sequencing technology to resequence the whole genomes of three parental and eighteen offspring samples, obtaining approximately 348.69 Gb of raw data, which was reduced to about 338.80 Gb after data cleaning. The filtered dataset for all samples was roughly 15 Gb, with an N50 length varying from 17 Kb to 32 Kb. The average read quality surpassed a score of 11, achieving a Q7 score of 100% (Supplementary Table S1). Alignment with the reference genome demonstrated a match rate exceeding 99%, with an average depth of approximately 9x (Supplementary Table S2). To maximize the replication of the female SVs and the accuracy of males and offspring SVs, we utilized multiple algorithms for PF and only Sniffles2 for males and offspring (Cuenca-Guardiola et al., 2023). Detection of PF’s SVs through tools such as Sniffles2, cuteSV, and SVIM resulted in 474,628, 1,012,709, and 87,059 SVs, respectively. These data were aggregated to represent SVs of PF (Table 1). Detection of SVs in males and offspring retained between 48.2% and 64.9% of the SVs after filtered (Supplementary Figure S2; Supplementary Table S3). CM showed a total of 492,833 SVs spanning 227.11 Mb, while PM and offspring ranged between 217,203 and 277,826 SVs spanning 246.25 Mb to 367.57 Mb (Figures 2, 3). DEL and INS were predominant in number, while INV and DUP, though fewer, were significant in total length, representing larger segment variations. The larger genomic sequence differences between the sea carp and the Pengze crucian carp are determined through the calculation of DSVs (Supplementary Table S4). CM with higher SV numbers but lower total length, indicating smaller fragment SVs. Subsequent DSVs detection showed CM had 44–67 times more SVs than PM and offspring (Figure 4; Supplementary Figure S3), reflecting genomic sequence evolution driven by its unique growth environment. PM and offspring exhibited consistency in the number and type of SVs, indicating genetic stability and limited changes in environmental adaptability.

**TABLE 1:** Quantitative SVs statistics across different software of PF.

Software	DEL	INS	INV	DUP	Total
Sniffles2	254,513	219,115	693	307	474,628
SVIM	491,203	515,673	1,132	3,958	1,012,709
cuteSV	45,968	41,001	50	40	87,059

### 3.2 Analysis and detection of DSVs and MSSVs in offspring

To detect DSVs within the genomes of male and offspring samples, we employed a combination of SURVIVOR and custom Python scripts. This involved iterative processes of merging the SVs detected in the PF with those of the offspring, followed by rigorous filtering to ensure that identified DSVs were unique and not present in PF. In the offspring, DSVs were detected in a range of 4,783–7,665, constituting approximately 2.19%–2.66% of their filtered SVs (Figure 4). The observed variation indicates the emergence of novel SVs, which may result from a variety of mechanisms. These include sporadic homologous recombination and high-frequency gene conversion during the maturation of oocytes (Wang et al., 2022), the suppression of DNA double-strand break formation and homologous recombination in oocytes (Lu et al., 2022), or the incorporation of male genetic fragments during the gynogenetic process (Zhang et al., 1999; Zhao et al., 2004a; Mao et al., 2020; Ding et al., 2021; Zhao et al., 2021). The process of recombination, depending on its accuracy, plays a dual role: inaccurate recombination can lead to the introduction of new genetic variations, whereas precise recombination has the potential to correct existing genetic errors (Burssed et al., 2022). Consequently, it is hypothesized that the DSVs observed in offspring may originate from both standard and aberrant recombination repair pathways, as well as directly from male genetic contributions.

**FIGURE 5:**
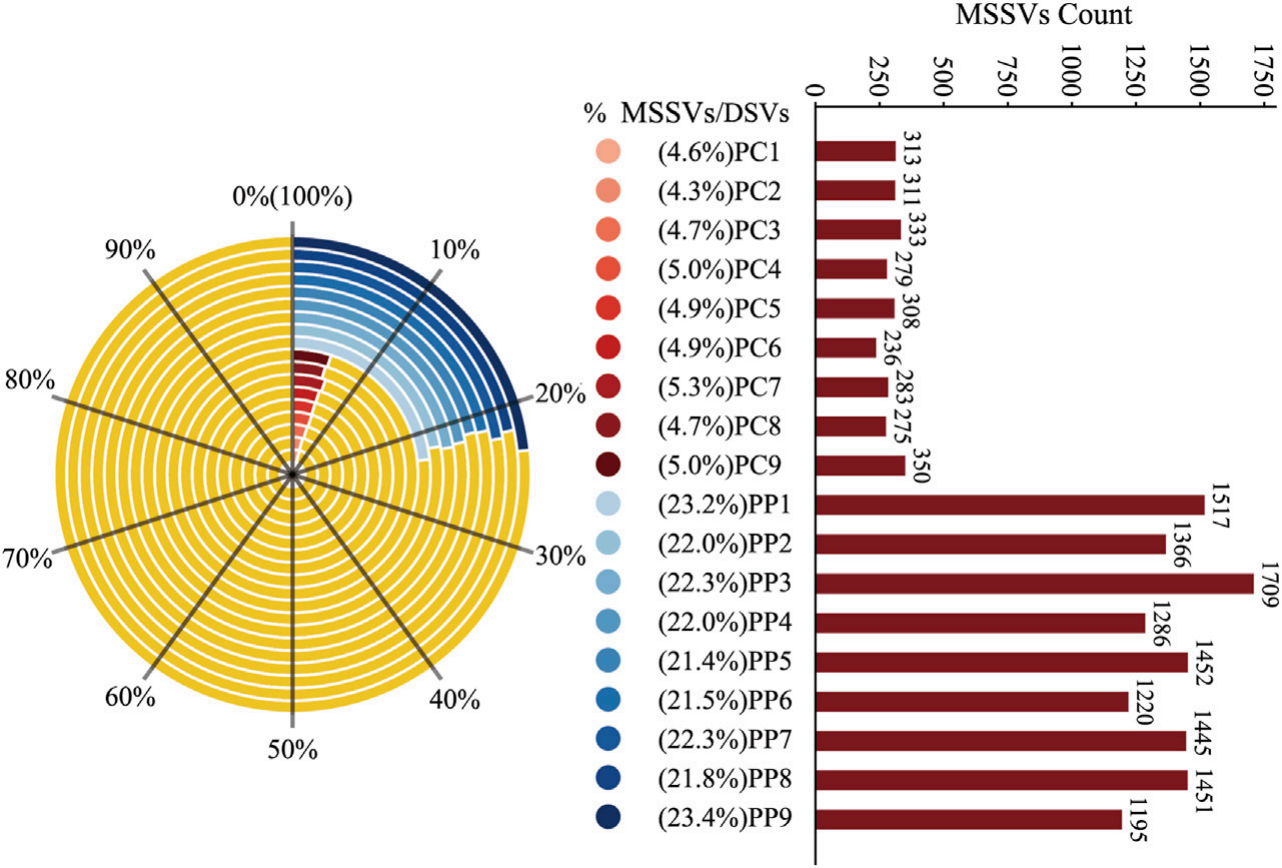
Proportion and Count of MSSVs in Homologous and Heterologous Offspring. The left side of the figure shows a radar chart depicting the percentage of MSSVs relative to DSVs in homologous (blue) and heterologous (red) offspring groups. The right side displays a bar graph with the absolute counts of MSSVs for each offspring, color-coded to match the radar chart segments.

In further analysis, we distinguished MSSVs in offspring by merging DSVs from homologous pairs with those from the PM and DSVs from heterologous pairs with the CM. Findings revealed that 21.4% to 23.4% of the DSVs in homologous offspring were MSSVs, with counts between 1,195 and 1709, while heterologous offspring had 4.3% to 5.0% MSSVs, with counts between 236 and 350 (Figure 5). This finding suggests that a significant proportion of the DSVs in the offspring can be traced back to male genetic contributions. Remarkably, even though the CM exhibited a DSV count that was 52 times higher than that of the PM (Supplementary Figure S3), the offspring derived from homologous sperm stimulation displayed a substantially higher prevalence of MSSVs—ranging from 3 to 7 times greater—compared to those conceived through heterologous sperm stimulation.

To identify common MSSVs across groups, we conducted a merged analysis for the nine homologous and nine heterologous offspring separately. In the gynogenetic triploid crucian carp, which possesses 150 basic chromosomes forming two sets of haplotypes (Wang et al., 2022), this analysis unveiled 14 common MSSVs across 11 chromosomes in homologous offspring (A4, A11, A12, A18, A22, A25, B1, B5, B14, B22, and B25) and only 4 common MSSVs across four chromosomes in heterologous offspring (A3, B14, B20, B21) (Supplementary Figure S4). This outcome suggests that the distribution of MSSVs is essentially random across chromosomes, without significant commonality between the two groups of offspring.

### 3.3 Annotation analysis of MSSVs in offspring

It is generally believed that MSSVs in offspring may have potential effects, we utilized the ANNOVAR tool for comprehensive gene annotation. Analysis showed that homologous offspring exhibited between 483 and 342 intergenic MSSVs, accounting for roughly 26.20% to 31.84% of their total MSSVs, and between 1,167 and 840 MSSVs in gene-associated regions, representing about 68.16%–73.80%. However, heterologous offspring presented with 58–90 intergenic MSSVs, making up approximately 21.53%–26.87%, and 180 to 277 MSSVs within gene-associated regions, equating to about 73.13%–78.47% (Supplementary Table S5).

**FIGURE 6:**
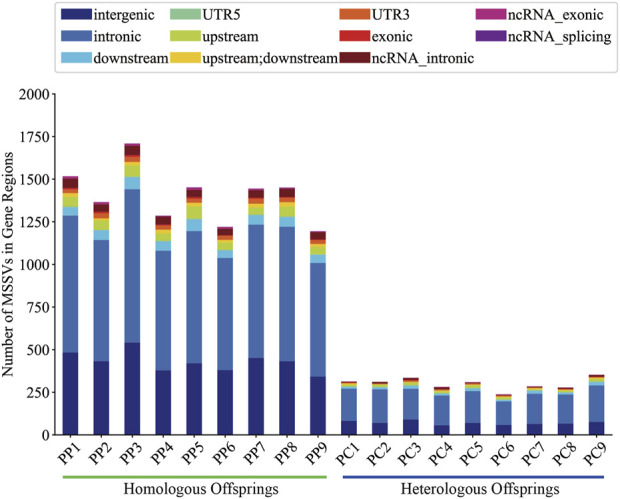
Localization of MSSVs in gene regions of homologous and heterologous offspring.

**FIGURE 7:**
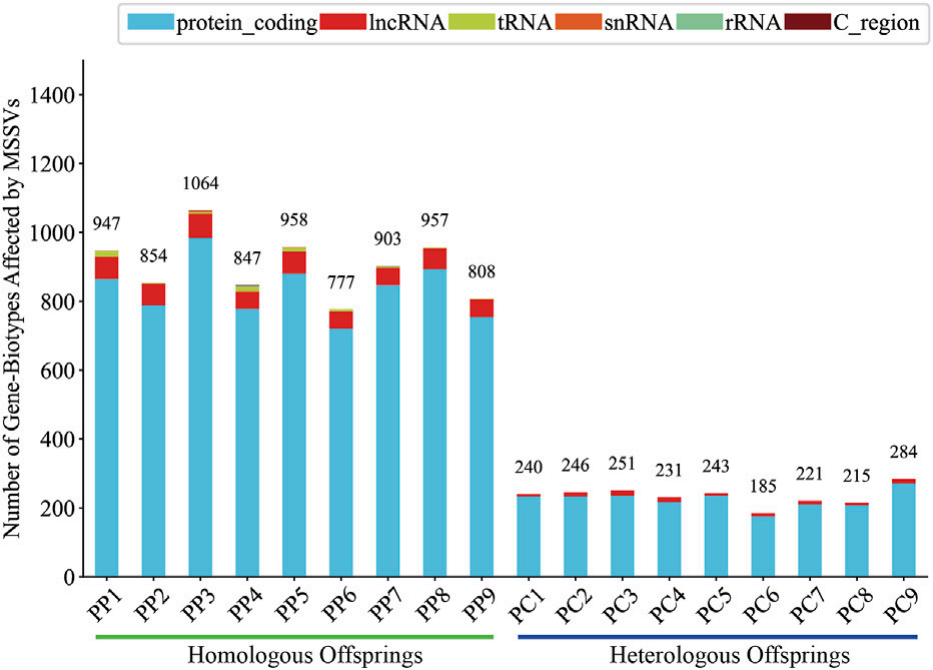
Count of gene-biotypes affected by MSSVs in homologous and heterologous offspring.

Generally, intergenic variations, located more than 2 Kb from known functional gene domains, are thought to exert minimal direct influence on gene functionality (Tropp, 2012). In contrast, MSSVs in proximate gene-associated regions (including upstream, downstream, exonic, UTR3, UTR5, ncRNA_intronic, ncRNA_exonic, and ncRNA_splicing areas) are positioned to significantly affect gene operation. The majority of MSSVs were identified in or near gene sequences (Figure 6), suggesting they could have considerable impacts, irrespective of the sperm stimulation method. Predominantly, impactful MSSVs were discovered within introns—non-coding segments known to contain sequences that might enhance gene expression (Rose, 2018). Following the exclusion of intergenic MSSVs, a detailed statistical analysis was conducted, revealing a significant number of genes affected in both homologous (777–1,064) and heterologous (185–284) offspring, primarily within protein-coding and lncRNA gene categories (Figure 7). This suggests these MSSVs predominantly affect biological types of gene like protein coding and lncRNA. This indicates a substantial influence of these MSSVs on crucial gene types, such as protein-coding and lncRNA genes, which are essential for protein synthesis and in regulating gene expression, epigenetics, and cell differentiation, respectively (Alberts et al., 2014; Quinn and Chang, 2016).

Distinctly, the annotation of common MSSVs varied between groups. In homologous offspring, among the 14 common MSSVs, distribution included 2 intergenic, 8 intronic, 2 upstream, 1 across both upstream and downstream, and 1 in UTR3 areas. For heterologous offspring, 4 common MSSVs were identified: 1 intergenic and 3 intronic (Supplementary Figure S5). Targeted analysis on exon region variations, crucial for encoding protein sequences and thus directly influencing protein structure and function, identified exon MSSVs in 6 genes among homologous offspring, all classified as INS. Notably, the sequence and position of these MSSVs in genes such as polyunsaturated fatty acid 5-lipoxygenase, transmembrane protease serine 13-like, and UNC93-like protein MFSD11 were consistent across several offspring (Table 2). While no universally conserved MSSVs were detected across all homologous offspring, specific exon MSSVs recurrently appeared in 3-4 offspring, hinting at a selective presence in distinct regions. In contrast, the identification of exon MSSVs in heterologous offspring was limited due to stringent filtering challenges.

**TABLE 2:** Statistical table of exons of genes affected by offspring MSSVs.

Offspring	Gene	Gene symbol	Mutation types	SV types	Chromosome	Position
PP1	polyunsaturated fatty acid 5-lipoxygenase	LOC128017081	frameshift substitution	INS	A7	28,699,668
PP2	uncharacterized LOC127957002	LOC127957002	nonframeshift substitution	INS	A3	6,374,385
	chondroadherin-like a	chadla	frameshift substitution	INS	B3	26,346,657
	transmembrane protease serine 13-like	LOC127957934	frameshift substitution	INS	B5	31,267,530
PP3	UNC93-like protein MFSD11	LOC127945018	frameshift substitution	INS	A3	7,334,819
	polyunsaturated fatty acid 5-lipoxygenase	LOC128017081	frameshift substitution	INS	A7	28,699,675
	transmembrane protease serine 13-like	LOC127957934	frameshift substitution	INS	B5	31,267,530
PP4	polyunsaturated fatty acid 5-lipoxygenase	LOC128017081	frameshift substitution	INS	A7	28,699,669
PP5	UNC93-like protein MFSD11	LOC127945018	nonframeshift substitution	INS	A3	7,334,823
	polyunsaturated fatty acid 5-lipoxygenase	LOC128017081	frameshift substitution	INS	A7	28,699,671
	transmembrane protease serine 13-like	LOC127957934	frameshift substitution	INS	B5	31,267,530
PP7	transmembrane protease serine 13-like	LOC127957934	frameshift substitution	INS	B5	31,267,531
PP8	UNC93-like protein MFSD11	LOC127945018	nonframeshift substitution	INS	A3	7,334,819

### 3.4 Validation of a specific MSSV in offspring

Confirming the reliability of detected MSSVs in offspring. We focused on validating an MSSV within the polyunsaturated fatty acid 5-lipoxygenase gene, chosen due to its significant mutation length and prevalence across multiple offspring samples. This mutation, situated on chromosome A7, constitutes a frameshift mutation that potentially alters the gene’s coding sequence, thereby modifying the amino acid sequence during protein synthesis (Table 2). Polyunsaturated fatty acid 5-lipoxygenase, a dioxygenase enzyme, plays a crucial role in catalyzing the conversion of polyunsaturated fatty acids, such as linoleic acid and arachidonic acid, into peroxides. This process is pivotal for initiating inflammatory responses and activating various cell death pathways, including apoptosis, ferroptosis, and necroptosis (Sun et al., 2019). While research on this gene’s function in fish remains sparse, its mutations are believed to significantly impact its enzymatic activity, potentially altering cell death mechanisms in offspring. The study of this gene in fish is currently limited, but its mutation can affect its function and consequently influence cell death patterns in offspring.

**FIGURE 8:**
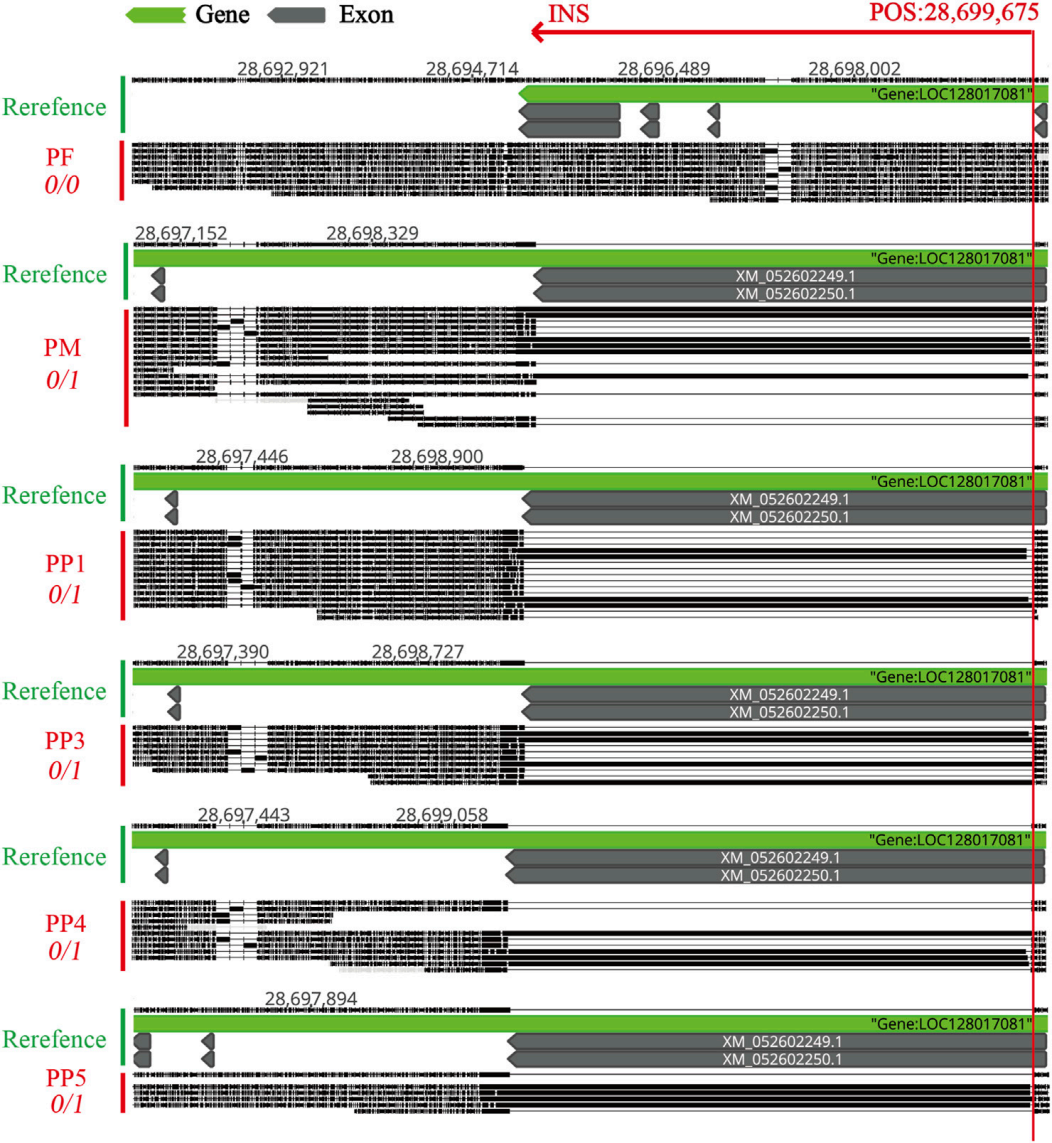
Comparative Visualization of the MSSVs INS in the Exon Region of the Polyunsaturated Fatty Acid 5-Lipoxygenase Gene Across PF, PM, PP1, PP3, PP4, and PP5. “POS: 28,699,675” indicates the physical insertion point of the INS on the reference chromosome. “0/0” means none of the reads support a mutation at this site. “0/1” means some reads support the presence of the mutation at this location.

**FIGURE 9:**
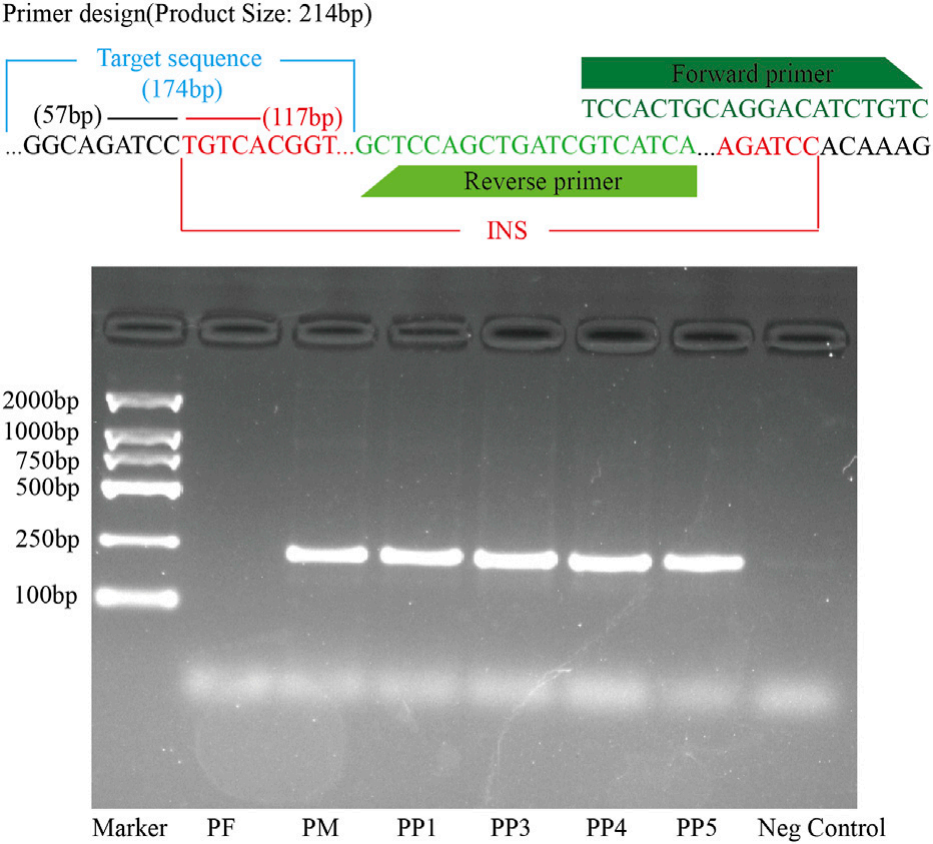
PCR Validation of the MSSV Insertion in the Exon Region of the Polyunsaturated Fatty Acid 5-Lipoxygenase Gene Across PF, PM, PP1, PP3, PP4, and PP5. The top panel displays the primer design specific for detecting the MSSVs. The target sequence, forward primer, and reverse primer are indicated. The square agarose gel electrophoresis image shows the results of the PCR amplification.

To accurately pinpoint the mutation’s insertion site, we utilized Geneious Prime for sequence alignment and correction. The insertion was identified at position 28,699,675, spanning a sequence length of approximately 5174 bp to 5210 bp (Figure 8; Table 2). The observed sequence length variability is likely due to the inherent error rates associated with sequencing technology and alignment processes. Notably, within PF sample, this mutation was absent, suggesting a homozygous state. Conversely, in PM and offspring samples PP1, PP3, PP4, and PP5, the mutation was supported by at least four reads, indicating heterozygosity. A striking match of four consecutive bases “AGATCC” at the end of the insertion sequence with the original sequence at the mutation site further substantiates this finding (Figure 9).

For further validation, primers were designed using Primer3, targeting both the region preceding the insertion point and the sequence immediately before the variant. Subsequent DNA electrophoresis revealed distinct bands in PM and offspring samples PP1, PP3, PP4, and PP5, but not in PF, unequivocally confirming the inheritance of this MSSV in a subset of the offspring (Figure 9; Supplementary Figure S6).

## 4 Discussion

Gynogenesis, a unique form of asexual reproduction, showcases distinct characteristics. Throughout the reproductive cycle, females produce eggs that are unreduced, carrying the same set of chromosomes as their somatic cells, and require sperm to act as a pseudogamete stimulus. This process exclusively utilizes female genetic material to initiate embryogenesis, leading to offspring that are genetically very similar to the female parent (Hubbs and Hubbs, 1932). In our investigation, through the application of a targeted strategy for the detection of SVs, we identified relatively low frequencies of DSVs and MSSVs in the Pengze Crucian Carp offspring, irrespective of whether homologous or heterologous sperm stimulation was employed. Specifically, homologous offspring presented with 5,116–7,665 DSVs (representing approximately 2.2%–2.7% of their detected SVs) and 1,195 to 1709 MSSVs (constituting about 0.52%–0.60% of their detected SVs). Meanwhile, heterologous offspring demonstrated 4,783 to 7,232 DSVs (approximately 2.2%–2.7% of their detected SVs) and 236 to 350 MSSVs (about 0.10%–0.13% of their detected SVs). Remarkably, the range of DSVs observed in offspring remained consistently within 2.2%–2.7%, regardless of the sperm stimulation method applied. Drawing on cytological evidence of sporadic homologous recombination and reduced double-strand break repair in oocytes (Lu et al., 2022; Wang et al., 2022), it appears that DSVs, rather than being male-specific, may primarily result from both accurate and inaccurate homologous recombination processes, whereas MSSVs are derived from male genetic contributions. Notably, under homologous sperm stimulation, the proportion of DSVs classified as MSSVs (21.4% to 23.2%) significantly surpassed that observed with heterologous sperm stimulation (4.3% to 5.3%).

These findings indicate that the genetic transfer and variation mechanisms within the gynogenetic reproductive mode of Pengze Crucian Carp are more intricate than previously comprehended. It is inferred that oocytes can generate SVs within a consistent range through either accurate or inaccurate homologous recombination, regardless of the sperm type used for stimulation. However, the presence of MSSVs is contingent upon whether the male contribution is homologous or heterologous, with homologous contributions exerting a greater genetic influence. Recent research, such as gynogenetic reproduction experiments in grass carp (*Ctenopharyngodon idellus*) using inactivated koi carp (*Cyprinus carpio haematopterus*) sperm, has unveiled recombined genes, like *HoxC6b*, resulting from the inheritance of male DNA (Mao et al., 2020)**.** Similarly, in natural gynogenetic silver carp, it was discovered that homologous male microchromosomes could be inherited by offspring, playing a pivotal role in enhancing offspring genetic diversity (Ding et al., 2021; Zhao et al., 2021). Furthermore, in gynogenetically reproduced offspring of the threespine stickleback (*Gasterosteus aculeatus*) using inactivated sperm, there was evidence of a minor, yet potentially significant, amount of male ‘leakage’ genetic material (Currey et al., 2023). These findings challenge Müller’s ratchet theory (Kondrashov, 1982), which posits that the accumulation of deleterious mutations in asexually reproducing organisms could lead to species extinction, underscoring the necessity to re-evaluate the genetic dynamics of asexual reproduction modes. In Pengze Crucian Carp’s reproduction, beyond homologous recombination and gene conversion, there is also the transmission of male chromosomal fragments that help eliminate deleterious mutations and enhance genetic diversity among the offspring. Moreover, the impact of homologous sperm surpasses that of heterologous sperm. However, other studies on fish have not identified evidence of male genetic fragments being inherited by offspring (Hou et al., 2016; Oral et al., 2017). We propose that the gynogenetic reproductive mode may vary across different fish species, leading to disparate outcomes. Specifically, in gynogenetic triploid crucian carp stimulated with heterologous sperm, a very low proportion of offspring results from unreduced fusion reproduction, where the offspring receive a complete set of chromosomes from the mother and a haploid set from the father (Zhao et al., 2004b; Lu et al., 2023). This phenomenon is seldom reported in other gynogenetic fish species. The mechanisms by which paternal-specific SVs manifest in offspring warrant further exploration.

In the study, we further explored the potential impact of MSSVs on the growth of offspring. Utilizing various sperm types to stimulate gynogenetic triploid crucian carp eggs, observed distinct growth effects across different offspring groups in other studies. One study found that at the end of the rearing experiment (180 days old) for homologous and heterologous offspring of Pengze Crucian carp, the average weight of heterologous offspring was 38.48% and 29.80% higher than that of homologous offspring, showing a significant growth advantage (Zhou et al., 2020). Another study on the rearing of homologous and heterologous offspring of gynogenetic Qihe Crucian carp for 90 days found that the final body weight, weight gain, and specific growth rate of homologous offspring were significantly higher than those of heterologous offspring (*p* < 0.01) (Shi et al., 2022). These observations underscore the significant role of male-contributed genetic material in influencing offspring development. Our analysis revealed that a substantial proportion of MSSVs in homologous offspring (approximately 68.16%–73.80%) and in heterologous offspring (about 73.13%–78.47%) were located within regions potentially impacting gene function significantly (including upstream, downstream, exonic, UTR3, UTR5, ncRNA_intronic, ncRNA_exonic, and ncRNA_splicing areas), with a predominance in intronic regions affecting primarily protein-coding genes. The growth effects observed under different sperm stimulation conditions present a dichotomy: heterologous offspring may exhibit accelerated growth compared to homologous counterparts and *vice versa* (Zhou et al., 2020; Shi et al., 2022). This divergence suggests the complexity of genetic influences on growth traits. Our findings propose that heterologous sperm’s genetic contribution to offspring is markedly less impactful than that of homologous sperm, potentially affecting gene functionality to a lesser extent. Consequently, if heterologous offspring outpace homologous offspring in growth, it might be attributed to the female inheritance of advantageous growth traits, with the negative impact of heterologous sperm being less significant. This scenario implies that heterologous offspring benefit from superior female traits, whereas the growth of homologous offspring could be constrained by the dominant genetic influence of the homologous male. Alternatively, a faster growth rate in homologous offspring could suggest that the homologous male possesses growth-enhancing genetic traits, significantly benefiting the offspring. This intricate interplay of genetic factors underscores the need for a deeper understanding of how male contributions influence offspring development in gynogenetic species.

Furthermore, our study delved into MSSVs within the exonic regions of protein-coding genes in offspring. SVs located in these regions often result in significant alterations to the gene’s expression—either by disrupting the transcript reading frame entirely or by causing the loss or addition of protein segments. Such changes hold considerable implications for both gene functionality and protein architecture (Ho et al., 2020). Specifically, we identified MSSVs in the exonic regions of five genes (polyunsaturated fatty acid 5-lipoxygenase, chondroadherin-like a, transmembrane protease serine 13-like, UNC93-like protein MFSD11, uncharacterized LOC127957002) exclusively in homologous offspring, but not in heterologous offspring. This is in contrast to other studies that have reported the presence of male inheritance in heterologous offspring (Mao et al., 2020). The reasons for this discrepancy may include a limited sample size, stringent filtering criteria, or variability in sequencing data quality, making it challenging to identify in heterologous offspring in the study. The occurrence of MSSVs in homologous offspring suggests the potential integration of male or recombined genotypes into critical genes of some offspring. Given the evolutionary conservation of exonic regions in protein-coding genes, we specifically validated the longest MSSV identified in the polyunsaturated fatty acid 5-lipoxygenase gene among homologous offspring. Our validation confirmed this MSSV’s presence in multiple samples (PP1, PP3, PP4, and PP5), emphasizing its significance. The polyunsaturated fatty acid 5-lipoxygenase gene plays a pivotal role in fatty acid metabolism and modulating cellular inflammatory responses (Sun et al., 2019). Similarly, chondroadherin-like a is implicated in chondrocyte proliferation and differentiation (Tillgren et al., 2014), while the transmembrane protease serine 13-like is crucial for activating hepatocyte growth factor and influencing cell signaling pathways (Hashimoto et al., 2010). Moreover, UNC93-like protein MFSD11 is instrumental in regulating energy metabolism and neural activities within both the central nervous system and peripheral tissues (Perland et al., 2016). Alterations within the exonic regions of these genes due to MSSVs could, therefore, significantly impact individual health and adaptability to environmental changes.

In our research, we leveraged cutting-edge third-generation long-read sequencing technology to identify SVs in gynogenetic Pengze crucian carp, shedding light on the substantial influence of male genetic contributions on offspring’s genetic diversity. This method proved especially effective in highlighting the dominant genetic influence of homologous sperm over heterologous sperm, a finding corroborated by PCR validation. When compared to methods like random amplified polymorphic DNA detection, microsatellite sequence polymorphism detection, and second-generation short-read sequencing for single nucleotide polymorphism identification, the precision and specificity of third-generation sequencing in pinpointing male-specific genetic fragments were unparalleled, mainly due to its ability to accurately identify long fragment SVs.

Nonetheless, our study is not without its limitations. It is important to acknowledge that our research may underestimate the rate of male genetic contribution, primarily due to the stringent filtering criteria employed. The actual impact could be greater than our statistical results suggest. An expanded sample size and increased sequencing depth would enhance the accuracy of our quantitative analyses, allowing for a more detailed examination of male genetic contributions and facilitating a probabilistic analysis of selected genetic regions. The primary challenge, however, remains the high costs associated with third-generation long-read sequencing technology, which significantly inflate the overall expenses of sequencing. Moreover, while we have identified MSSVs in offspring, the underlying mechanisms and their potential functional consequences remain largely unexplored. Future research should pivot towards understanding the functional implications of these MSSVs, particularly their impact on the phenotypic traits and adaptability of offspring. Delving into the processes through which selective male genetic material is incorporated into gynogenetic organisms will be paramount. Extending this research to other gynogenetic species could unveil whether similar genetic phenomena are prevalent, thereby enriching our comprehension of the mechanisms underpinning asexual reproduction.

## Data Availability

The datasets supporting the conclusions of this article are available in the NCBI BioProject repository, under the unique persistent identifiers PRJNA1048717 (https://www.ncbi.nlm.nih.gov/bioproject/PRJNA1048717) and PRJNA1070022 (https://www.ncbi.nlm.nih.gov/bioproject/PRJNA1070022/).
