# Supplementary figures and images for: Deciphering male influence in gynogenetic Pengze crucian carp (Carassius auratus var. pengsenensis): insights from Nanopore sequencing of structural variations
Source: Front Genet. 2024 May 9;15:1392110. doi: 10.3389/fgene.2024.1392110 (PMC11111978; doi:10.3389/fgene.2024.1392110)

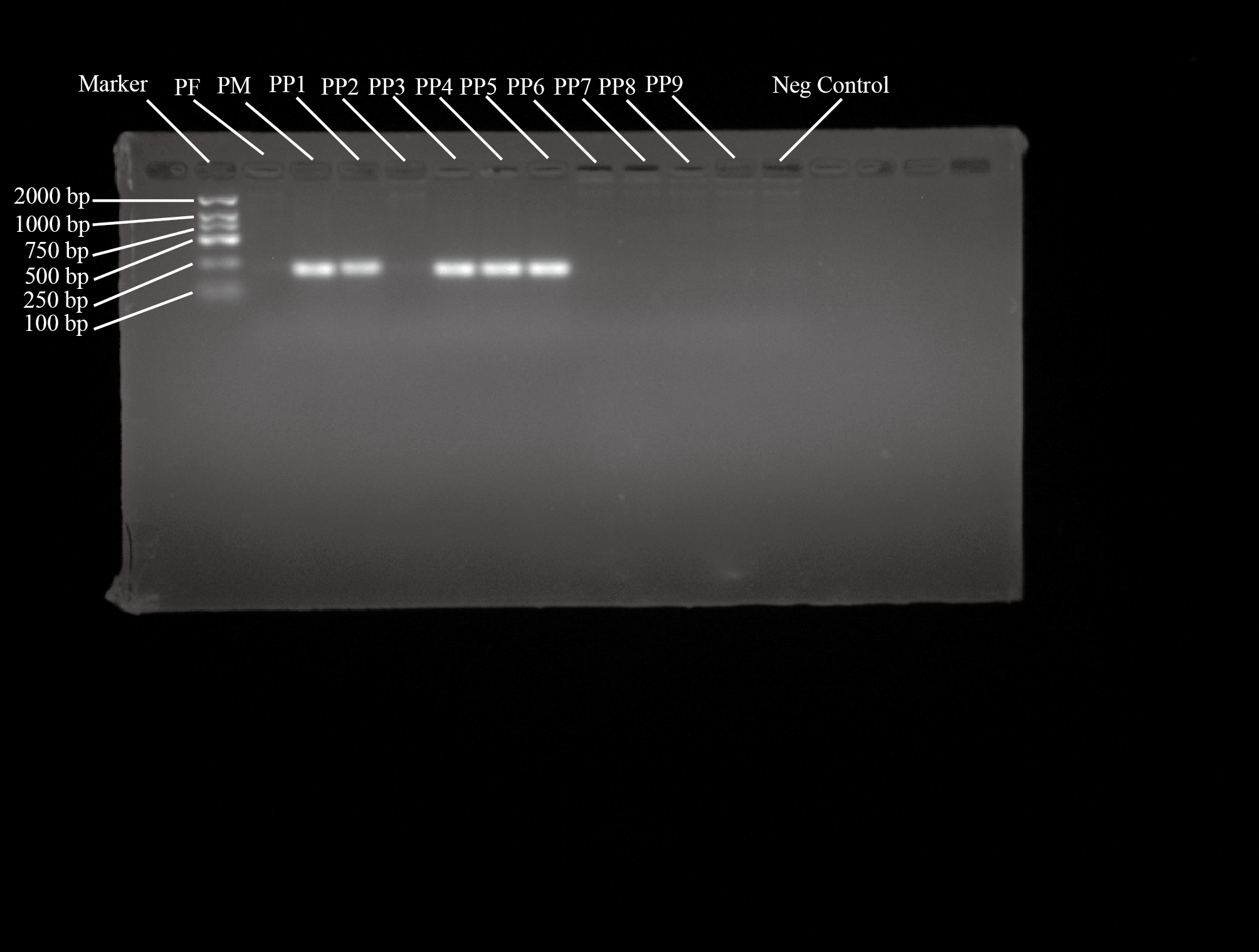

Supplement: Supplementary file 2 [file Image6.TIF]

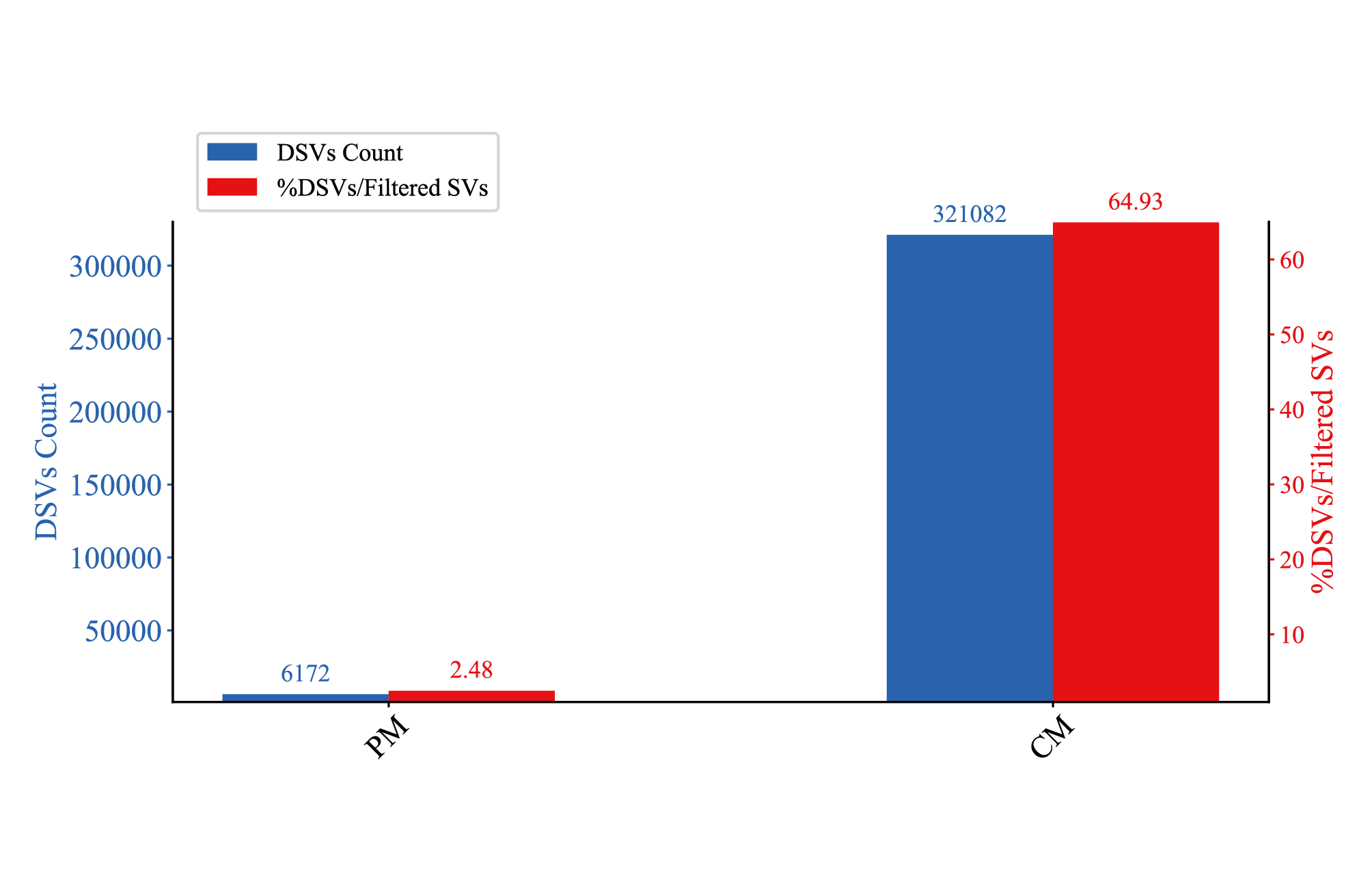

Supplement: Supplementary file 3 [file Image3.TIF]

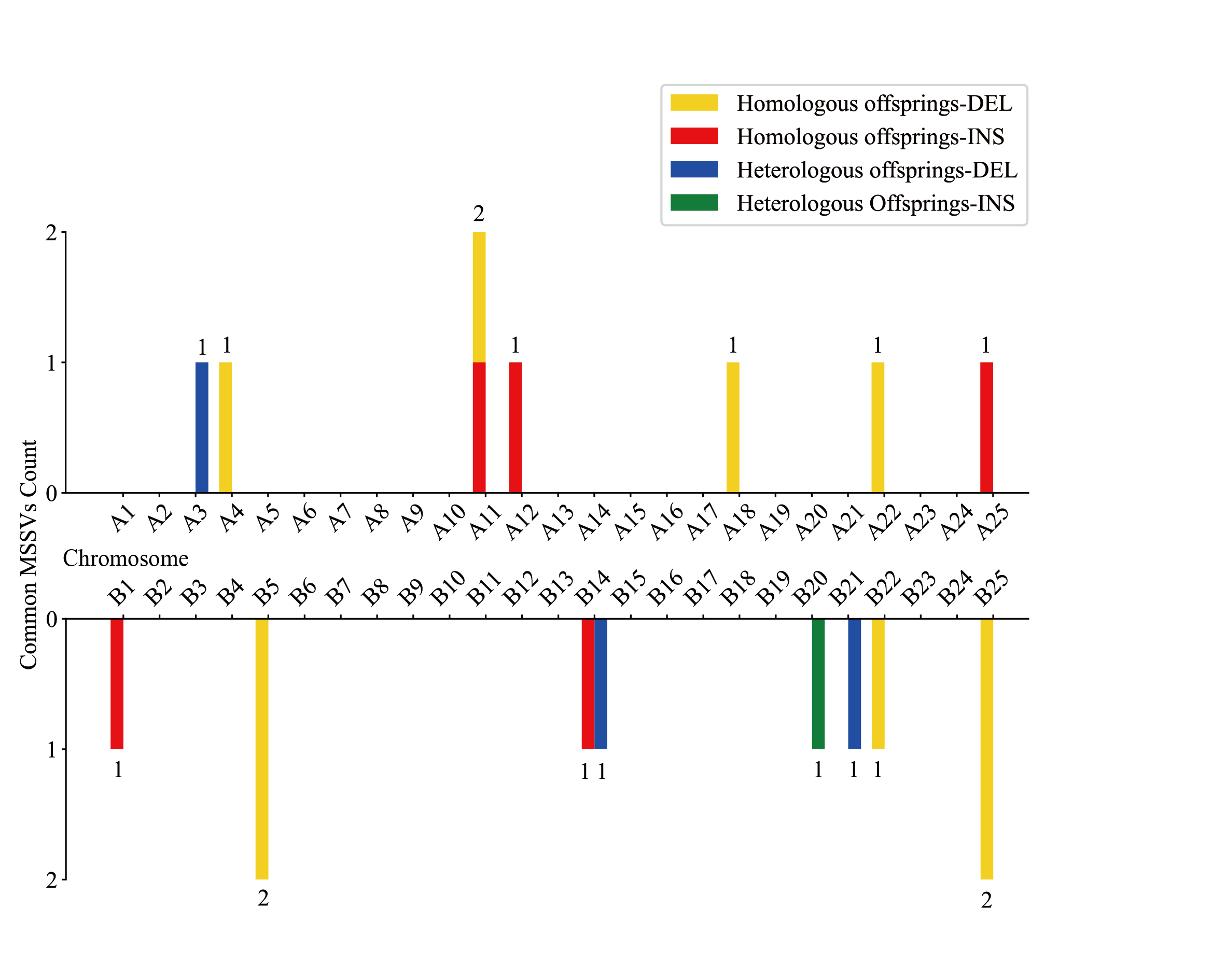

Supplement: Supplementary file 4 [file Image4.TIF]

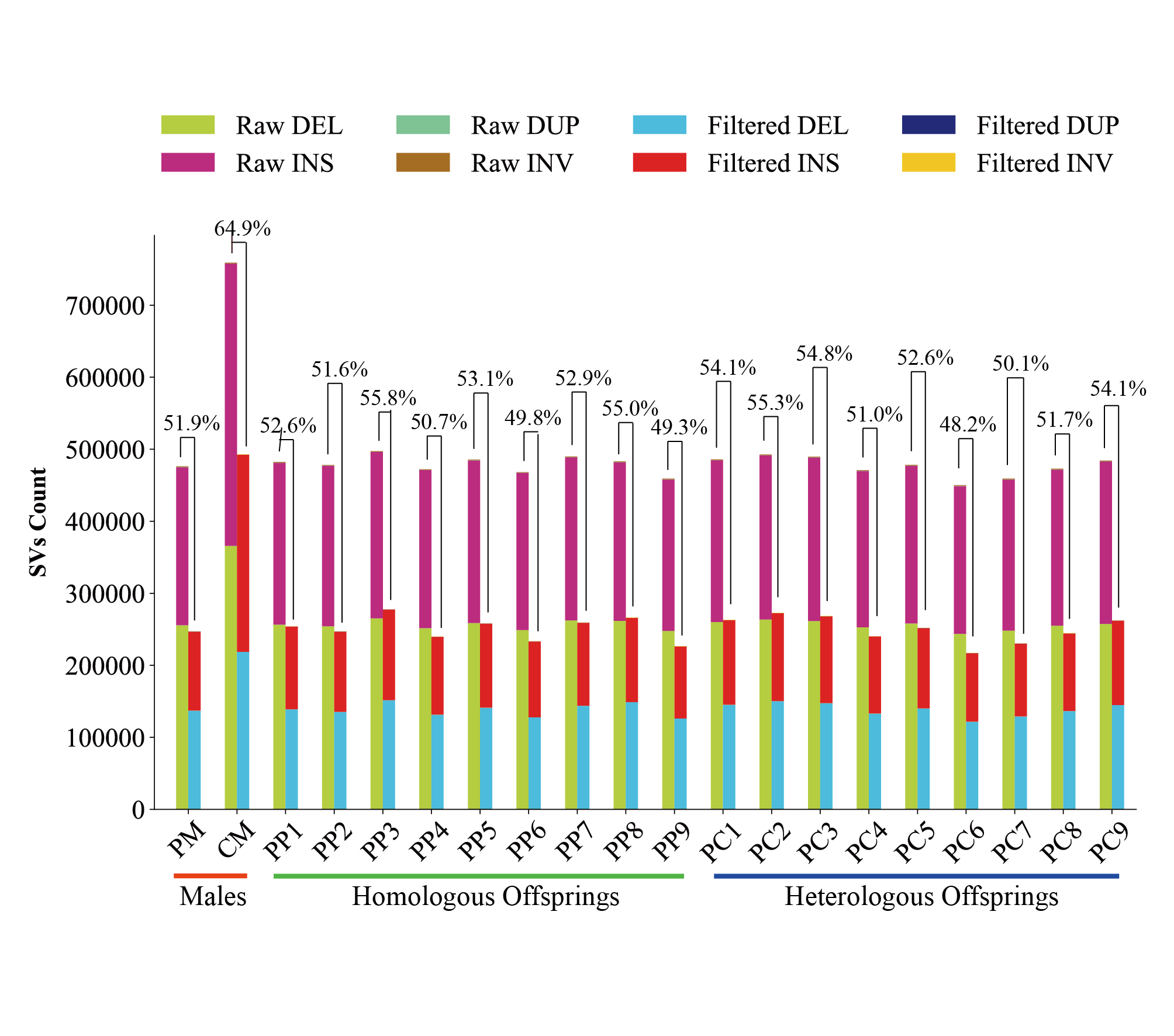

Supplement: Supplementary file 5 [file Image2.TIF]

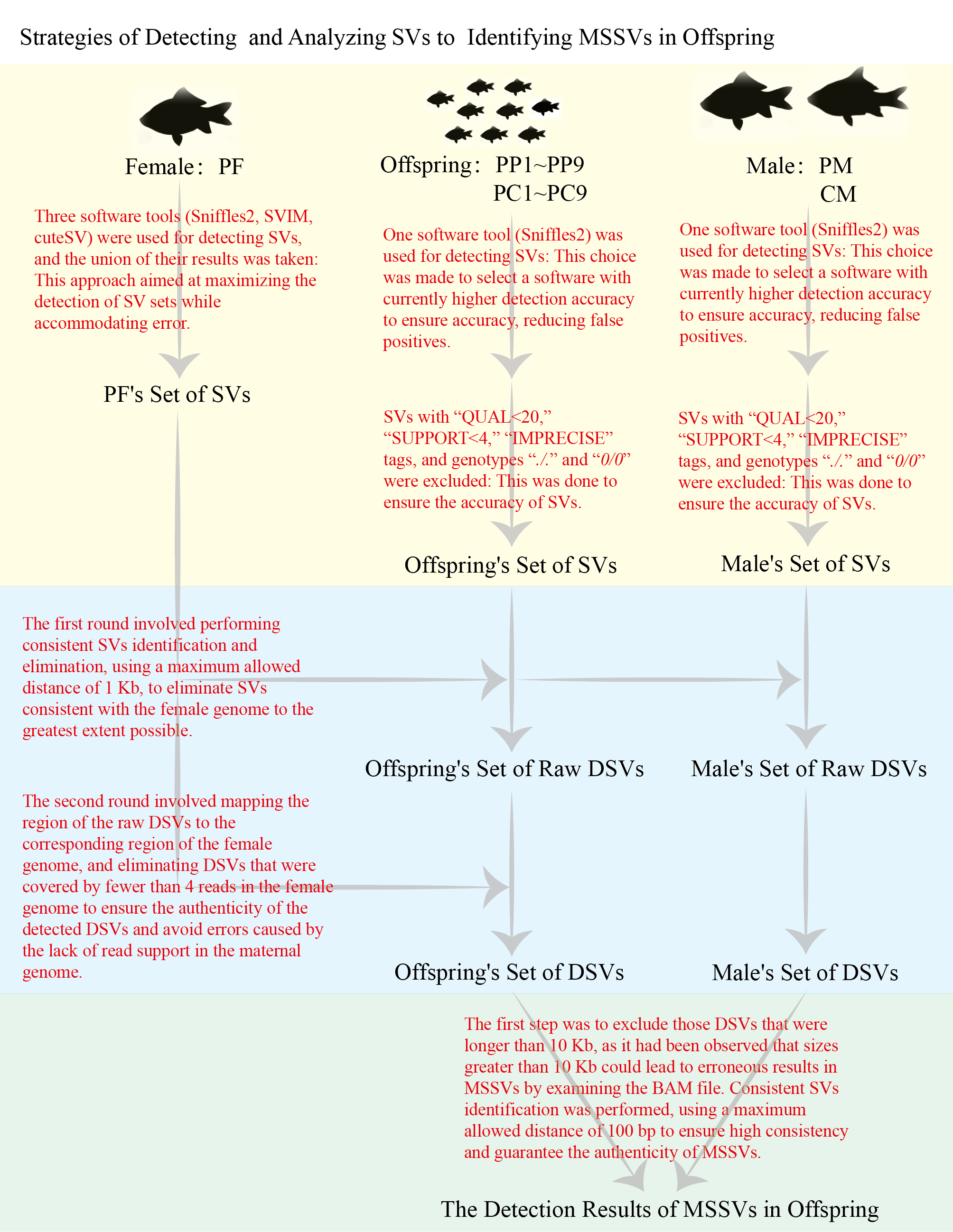

Supplement: Supplementary file 6 [file Image1.TIF]

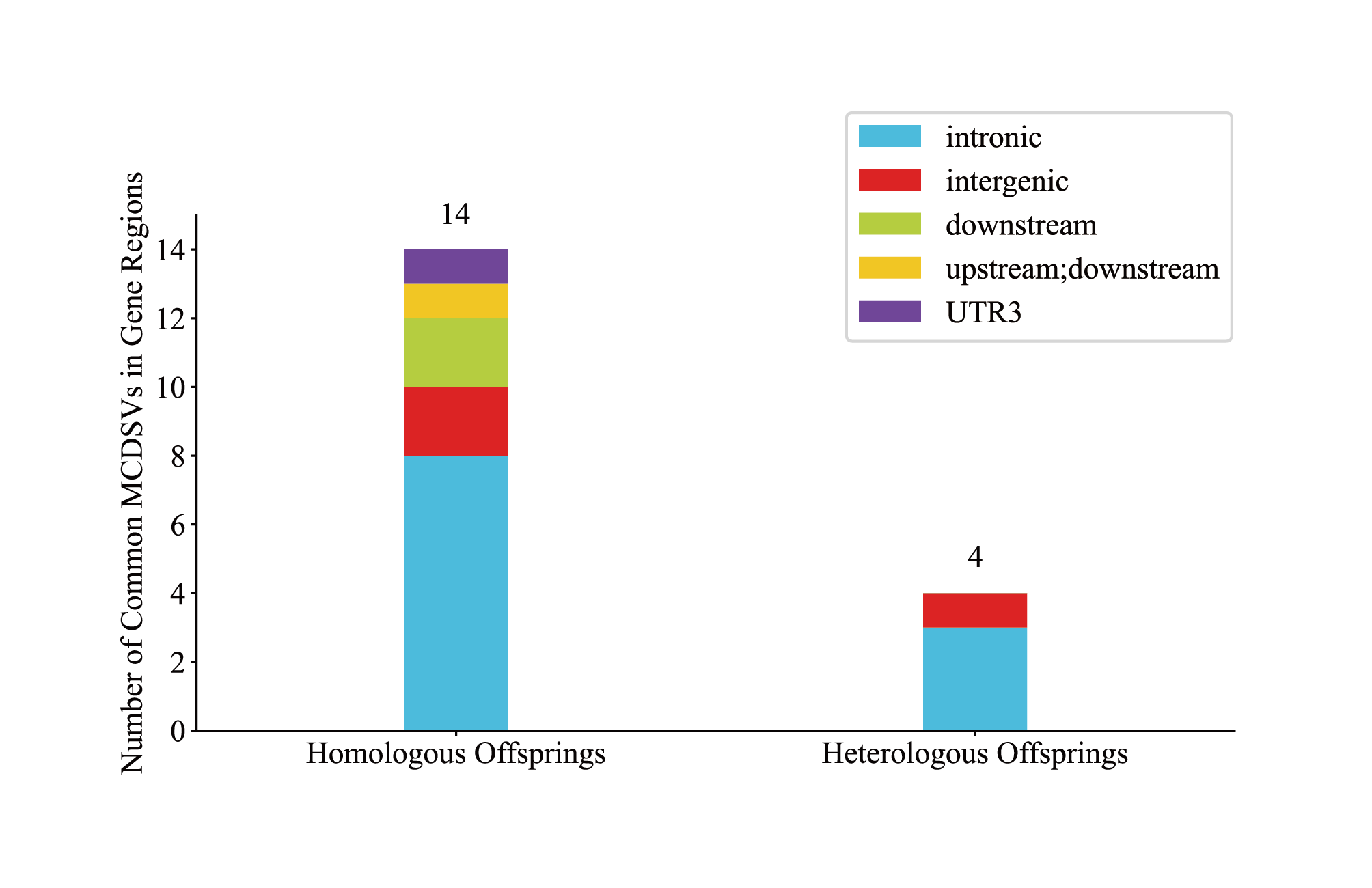

Supplement: Supplementary file 10 [file Image5.TIF]
