# Supplementary material for: Deciphering male influence in gynogenetic Pengze crucian carp (Carassius auratus var. pengsenensis): insights from Nanopore sequencing of structural variations
Source: Front Genet. 2024 May 9;15:1392110. doi: 10.3389/fgene.2024.1392110 (PMC11111978; doi:10.3389/fgene.2024.1392110)
Supplement: Supplementary file 7 [file Table9.DOCX]

**Table of Contents**

**Figure S1.** **Strategies of Detecting and Analyzing SVs to Identifying MSSVs in Offspring.**

**Figure S2. Filtration and Type Distribution of SVs in Males, Homologous, and Heterologous Offspring.** The percentages above the bars indicate the proportion of SVs retained after filtration.

**Figure S3. DSVs Count and Proportion in PM and CM.** The bar chart compares the absolute count of DSVs (blue bars) identified in PM and CM. The red bars represent the percentage of these DSVs relative to the total filtered SVs for PM and CM.

**Figure S4.** **Chromosomal Distribution of Common MSSVs in Homologous and Heterologous Offspring.**

**Figure S5.** **Localization of Common MSSVs in Gene Regions of Homologous and Heterologous Offspring.**

**Figure S6. PCR Validation of the MSSV Insertion in the Exon Region of the Polyunsaturated Fatty Acid 5-Lipoxygenase Gene Across PF, PM, PP1~PP9.**
